# Hematopoietic differentiation at single-cell resolution in *NPM1*-mutated AML

**DOI:** 10.1038/s41408-022-00734-1

**Published:** 2022-09-23

**Authors:** Matthieu Duchmann, Romane Joudinaud, Augustin Boudry, Justine Pasanisi, Giuseppe Di Feo, Rathana Kim, Maxime Bucci, Clémentine Chauvel, Laureen Chat, Lise Larcher, Kim Pacchiardi, Stéphanie Mathis, Emmanuel Raffoux, Lionel Adès, Céline Berthon, Emmanuelle Clappier, Christophe Roumier, Alexandre Puissant, Claude Preudhomme, Nicolas Duployez, Raphaël Itzykson

**Affiliations:** 1 Université Paris Cité, Unité 944/7212-GenCellDi, INSERM and Centre National de la Recherche Scientifique (CNRS), Paris, France; 2 Hematology Laboratory, Unité 1277-Cancer Heterogeneity Plasticity and Resistance to Therapies (CANTHER), Centre Hospitalier Universitaire (CHU) de Lille, University of Lille, Institut National de la Santé et de la Recherche Médicale (INSERM), Lille, France; 3 Hematology Laboratory, Saint Louis Hospital, Assistance Publique-Hôpitaux de Paris (AP-HP), Paris, France; 4 Hematology Department, Saint Louis Hospital, AP-HP, Paris, France; 5 Hematology Department, Centre Hospitalier Universitaire (CHU) de Lille, University of Lille, Lille, France

**Keywords:** Translational research, Acute myeloid leukaemia

Dear Editor,

Recent data suggest that *NPM1-*mutated AMLs are heterogeneous in terms of co-mutations and expression of transcriptomic programs and surface proteins [1, 2]. Such heterogeneity may reflect a variable differentiation blockade of leukemic cells. Indeed, blasts in some *NPM1*-mutated AMLs may display an immature progenitor morphology, immunophenotype, and transcriptional program, or have a more mature monocytic and/or dendritic differentiation in other patients [1–3]. These differences can be clinically important, as immature blasts might have higher stemness capacity, a feature associated with poorer outcomes in AML [4]. Conversely, blasts with monocytic differentiation may have immunosuppressive capacities, and relative BCL-2 independence [5]. Previous reports based on bulk sequencing revealed only weak associations between *NPM1/FLT3-ITD* genotype and immature phenotype and between *NPM1/FLT3-TKD* or *NPM1/RAS* genotypes and monocytic/dendritic differentiation [2, 3]. Novel technologies allow simultaneous genotyping and analysis of surface protein expression at single-cell resolution and may help to resolve the interconnection between genotype and cell differentiation in leukemia [6, 7]. We used a droplet-based multi-omics single-cell platform to characterize the genetic clonal architecture in eleven *NPM1*-mutated AML diagnostic samples and investigate the relationship between co-mutations and phenotypic hematologic differentiation at the single-cell level.

We retrospectively included viably frozen samples from 11 patients with *NPM1*-mutated AMLs diagnosed in Saint Louis or Lille university hospitals banked after informed consent between May 2016 and July 2019. The project was approved by INSERM IRB (CEEI-20-274). Samples were selected if they had an *NPM1* mutation and at least two additional mutations covered by the Mission Bio AML amplicons panel. Molecular information was available from routine bulk high-throughput sequencing (HTS) using previously published custom capture panels at Saint Louis (*n* = 8, Table S1) or Lille (*n* = 3, Table S2) university hospitals [8]. Cryopreserved mononucleated cells were thawed and live cells were stained using a 15 antibodies derived tags (ADT) panel (Fig. 1A and Table S3). Cells were processed according to the manufacturer protocol, using Mission Bio 20-genes AML amplicon panel (Table S4). Libraries were sequenced on a Novaseq 6000 (Illumina). Fastq files were analyzed using Mission Bio Tapestri Pipeline V2 (Fig. 1B). Filt3R was used for *FLT3-ITD* detection. sc-DNAseq analysis was focused on variants also detected on bulk HTS, using the *TapestriR* package. A genotype was considered informative if the single-cell sequencing depth (scDP) was ≥10x. An allele was retained if supported by at least 3 reads, and a single-cell variant allelic frequency (scVAF) ≥15% for an scDP between 20–100× or ≥10% for an scDP >100x, or considered non-informative otherwise. infSCITE was used to infer phylogenetic trees from mutation matrices as published [7]. Inferred clonal architectures (Fig. S1A) were used to correct raw cell genotypes. Cells with insufficient genotype information or with a genotype violating the clonal hierarchy owing to cell doublets or sequencing errors were excluded for downstream analyses (Fig. S1B). Protein data was analyzed using Seurat package V4.0 [9]. ADT counts were transformed using centered log-ratio transformation and differential expression was tested using ALDEX2 [10]. Bulk and single-cell DNA-Seq will be available at European Genome-phenome Archive (EGA) under accession code EGAS00001006565. Other data will be available upon reasonable request to the principal investigator.

**Fig. 1:**
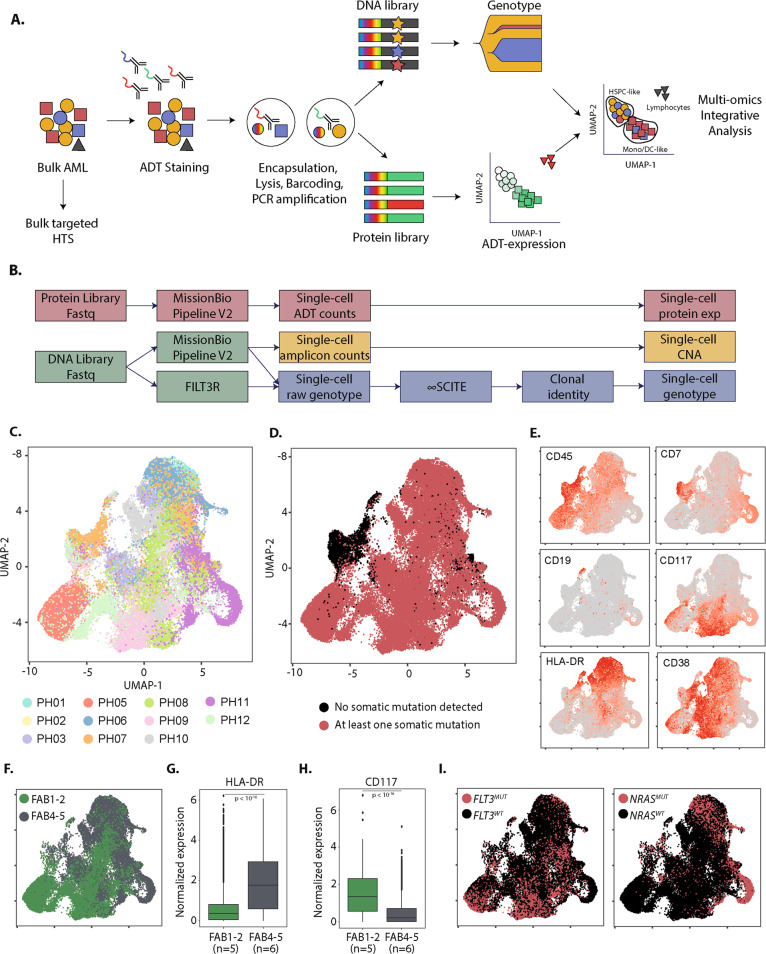
Single-cell multi-omics workflow and application to 11 NPM1-mutated AMLs. **A** Overview of the single-cell multi-omics platform. AML acute myeloid leukemia; HTS high-throughput sequencing; ADT antibody-derived tags. **B** Overview of the bioinformatics analyses performed on sequencing data. CAN copy number alterations. **C** Uniform manifold approximation and projection (UMAP) plot of ADT-seq expression of the 52,103 single-cells from the 11 AML samples. Cells are colored by sample. **D** UAMP plot of ADT-seq expression of the 52,103 single-cells from the 11 AML samples. Cells are colored according to the presence (red) or absence (black) of at least one somatic mutation. **E** UAMP plot of ADT-seq expression of the 52,103 single-cells from the 11 AML samples. Cells are colored according to specific ADT expressions. **F** UAMP plot of ADT-seq expression of the mutated cells from the 11 AML samples. Cells are colored according to the FAB classification at diagnosis: FAB1-2 (green) or FAB4-5 (gray). **G** Expression of HLA-DR ADT by mutated cells according to the FAB classification at diagnosis. **H** Expression of CD117 ADT by mutated cells according to the FAB classification at diagnosis. **I** UAMP plot of ADT-seq expression of the mutated cells from the 11 AML samples. Cells are colored according to the presence (red) or absence (black) of *FLT3* (left) or *NRAS* (right) mutations. *P* values from *t*-tests were corrected using the Benjamini & Hochberg method.

Characteristics of the 11 *NPM1*-mutated AML diagnostic samples are summarized in Table S5. A total of 61 mutations were detected by bulk HTS (Tables S5, 6), with a median number of five mutations per patient [range: 3–8]. The most frequently mutated genes were *FLT3* (9/11, 82%), *NRAS* (5/11, 45%), *DNMT3A* (4/11, 36%), *IDH2* (4/11, 36%), and *PTPN11* (4/11, 36%). Most patients (91%) had multiple signaling mutations, with a median number of three per patient [range: 1–5].

To correct the noise of sc-DNAseq data on ill-covered genomic regions, we developed a framework to perform phylogeny-driven genotype correction (Fig. S1A, B). This strategy increased the number of cells with complete genotype information from 34,722 to 52,103 single cells (Fig. 1C and S1C). The median number of cells per sample was 5123 [range: 2301–7127]. Most somatic mutations identified by bulk HTS (55/61, 90%) were detected by sc-DNAseq. Three patients had one mutation not covered by the sc-DNAseq panel, and one patient had two. These mutations affected *SRSF2* (*n* = 2), *RAD21* (*n* = 1), *SMC3* (*n* = 1), and *NFE2* genes (*n* = 1, Tables S5, 6). One covered *FLT3-ITD* was not detected by sc-DNAseq owing to its long size (102 bp). Our genotype correction strategy did not bias the data as pseudo-bulk VAF from sc-DNAseq were highly correlated to bulk HTS VAF (Spearman rho = 0.95, *p* < 10^−16^, Fig. S2A). Two patients had one homozygous mutation, and both bulk HTS and sc-DNAseq showed a copy-neutral loss of heterozygosity of *FLT3-ITD* in PH01 (Fig. S2B) and a deletion of the wild-type allele of *TET2* in PH05 (Fig. S2C). In keeping with previous findings, intra-leukemic genetic heterogeneity was detectable in all cases (Fig. S2D) [6, 7]. Clonal branching was detectable in nine patients. One additional patient (PH12) would presumably also have had a parallel evolution of the two *FLT3-ITD* clones if both had been detected. All cases of branched architectures involved co-occurring signaling mutations, as previously reported in other AML subtypes [6, 7].

Upon dimensional reduction on ADT-seq data across all samples, some degree of patient-centric clustering was noticeable, owing to specific combinations of surface markers (Fig. 1C). As expected in *NPM1*-mutated AMLs, leukemic cells did not express CD34 [3]. ADT-seq was able to discriminate normal lymphoid cells from all patients, as they did not harbor somatic mutation (Fig. 1D) and expressed high levels of CD45 with either CD7 or CD19 expression (Fig. 1E). As previously reported on flow cytometry analyses [1–3], ADT-seq was able to discriminate the 6 patients with a predominant morphologic myelomonocytic differentiation (FAB4-5) from the 5 patients with poorly differentiated cytology (FAB1-2, Fig. 1F). Leukemic cells from FAB4-5 patients had a higher expression of HLA-DR (*p* < 10^−16^, Fig. 1G) and a lower expression of CD117 (*p* < 10^−16^, Fig. 1H). Larger ADT panels, the addition of UMIs [10], and control isotypes will likely further improve the resolution of ADT-seq in single-cell multi-omics platforms.

We identified various degrees of intra-leukemic immunophenotype heterogeneity, as previously suggested [3]. Specific associations between surface proteins expression and somatic mutations were patient-specific (Fig. S3) rather than shared across patients (Fig. 1I), and larger studies will be requested to analyze cohort-wide genotype-phenotype correlations. Patient PH11 is shown as an illustrative case (Fig. 2). Unsupervised clustering of single cells based on ADT-seq revealed a marked clustering of cells harboring the *FLT3* p.A680V mutation (Fig. 2A). *FLT3-*mutated cells had higher expression of CD45, CD38, and HLA-DR and lower expression of CD117 compared to other leukemic cells (Fig. 2B–D), a phenotype reminiscent of monocytic differentiation. Conventional multiparametric flow cytometry confirmed the presence of distinct leukemic cell subpopulations. Bulk HTS of the FACS-sorted monocyte-like subpopulation (intermediate SSC, high CD45, HLA-DR+, CD38+, CD117−, CD13−, CD7−, Fig. 2E) confirmed the enrichment for *FLT3*-mutated cells (VAF of 40 vs 6% in bulk) at the expense of the *NRAS* and *GATA2* mutated clones (Fig. 2F and Table S7). This result comforts previous studies showing genetic differences between phenotypically distinct leukemic cell populations in individual patients [11]. To our knowledge, this is the first validation of ADT-seq results on sorted subpopulations defined by a specific combination of multiple cell-surface markers. Further validation could be conducted, e.g., using genetically engineered cell lines. The development of ADT panels larger than the first-generation 15-protein panel used in our study may alleviate the limitation of the compositional structure of small ADT panels. Further technological improvements, such as the addition of UMIs in ADT sequencing to remove PCR duplicates [10] and spiking of control isotypes to estimate unspecific binding and define positivity thresholds, will likely further improve the resolution of surface protein expression analysis in single-cell multi-omics platforms. Recent clonal phylogenies inference and single-cell protein expression normalization tools will also need to be benchmarked across various single-cell proteogenomic datasets [12]. Clone-specific immunophenotypes might allow the study of clone-specific drug sensitivity in phenotype-based ex vivo drug screening experiments [13–15]. Our study paves the way for single-cell multi-omics deciphering of the genetic and non-genetic contributions to differentiation blockade in AML and provides a proof of concept for precision oncology instructed by single-cell resolution of the genetic and phenotypic diversity of leukemic cells.

**Fig. 2:**
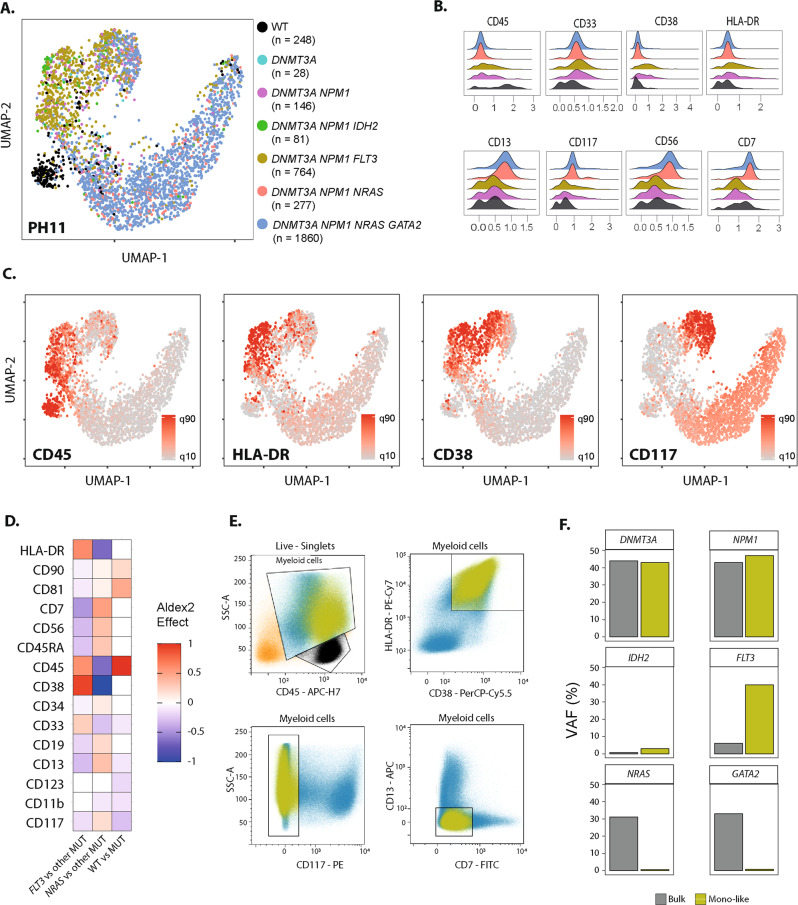
Clone-specific immunophenotypes in an NPM1-mutated AML. **A** UMAP plot of ADT-seq expression of the 3,404 single-cells from sample PH11. Cells are colored according to the genotype. **B** Ridge plots showing CLR-transformed expression of eight ADT according to the genotype of the cell from the PH11 sample (only genotypes with >100 cells are displayed). **C** UMAP of ADT-seq expression of the 3404 single-cells from sample PH11. Cells are colored according to the CLR-transformed expression of four ADT markers. **D** Differentially expressed ADT between *FLT3*-mutated and other cells harboring at least one mutation (left), *NRAS*-mutated and other cells harboring at least one mutation (middle), cells with at least one mutation, and cells without any mutation (right). The size of the difference is evaluated by the Effect parameter of Aldex2, which is displayed only for differentially expressed ADT (adjusted *p* value <0.05). **E** Gating strategy for FACS sorting monocyte-like leukemic cells (Boolean: Myeloid cells and HLA-DR+ and CD38+ and CD117− and CD7− and CD13−). Lymphocytes are colored black, monocyte-like cells are colored yellow, and other leukemic cells are colored blue. **F** Variant allelic fractions of the specified mutations on leukemic bulk (gray) and the sorted monocyte-like population (yellow).

## Supplementary information

Supplemental Appendix

## Data Availability

Bulk and single-cell DNA-Seq will be available at European Genome-phenome Archive (EGA) under accession code EGAS00001006565. Other data will be available upon reasonable request to the principal investigator.
