# Supplementary material for: Hematopoietic differentiation at single-cell resolution in NPM1-mutated AML
Source: Blood Cancer J. 2022 Sep 23;12(9):136. doi: 10.1038/s41408-022-00734-1 (PMC9508105; doi:10.1038/s41408-022-00734-1)
Supplement: Supplementary file 1 — Supplemental Appendix [file 41408_2022_734_MOESM1_ESM.docx]

**Supplemental appendix**

**Hematopoietic differentiation at single-cell resolution in *NPM1*-mutated AML**

Matthieu Duchmann*, Romane Joudinaud* et al.

**Supplementary Methods**

**Supplementary Figure legends.**

**Supplementary Figure S1-S3.**

**Supplementary Tables 1-7**

**Supplementary Material**

***Single-cell DNA-seq + phenotype sequencing***

Cryopreserved BMMC or PBMC were thawed and dead cells and debris were removed using magnetic beads (Dead cell removal, Miltenyi Biotec). Cells were stained using a custom panel of 15 antibodies (CD45, CD34, CD38, CD7, CD19, CD123, CD117, CD45RA, CD90, CD81, HLA-DR, CD56, CD11b, CD13, CD33, Biolegend) conjugated with specific DNA-oligonucleotides (**Figure 1A,** **Table S3)** for 30 minutes at room temperature. After three washes in PBS + 10% fetal bovine serum (FBS, Sigma), cells were resuspended in cell buffer (Mission Bio) and diluted to 3,500 cells/µL. Then, 35µL of cell suspension were loaded in a microfluidic cartridge for encapsulation, lysis and barcoding following Mission Bio protocol. Forward primers for protein library were added at the barcoding step (IDT, GTACTCGCAGTAGTCCGACTGAGATACTAGATCGGCTC, 2µL of 30µM). For genomic DNA library, we used a commercially available AML panel including 127 amplicons covering 20 genes recurrently mutated in AML (**Table S4**). A first PCR amplification was performed in emulsion with 24 cycles. Emulsions were then broken and DNA and protein libraries were purified using Ampure XP beads (Beckman Coulter) and custom biotinylated oligos (/**5Biosg** /CGAGATGACTACGCTACTCATGG/**3C6**/, IDT) respectively. Purified amplicons were indexed and amplified with a second PCR for 10 (DNA) and 20 (Protein) cycles. Libraries were finally purified using Ampure Beads, pooled and sequenced on a Novaseq 6000 (Illumina) at Lille university hospital’s sequencing facility.

***Bioinformatics analysis***

Bcl2 files were then demultiplexed using bcl2fastq. The bioinformatic analysis strategy is summarized in **Figure 1B**. Briefly, fastq files were analyzed using the Tapestri pipeline V2 for pre-processing, alignment, cell barcode correction, cell identification, variant calling, genomic DNA amplicons and antibody-derived tags (ADT) counting. Analysis was focused on variants also detected on bulk HTS. Bulk and single-cell bam files were also analyzed using the Filt3R algorithm that allows the robust detection and quantification of *FLT3-ITD.*^1^ Multiomics h5 files were then analyzed on R (version 4.0) using the *TapestriR* package. A genotype was considered for a genomic position if the single-cell sequencing depth (scDP) was ≥ 10x. An allele was retained if it was supported by at least 3 reads, and if the single-cell variant allelic frequency (scVAF) was ≥ 15% for a scDP between 20-100x, or a scVAF ≥ 10% for a scDP > 100x. The genotype was otherwise considered as missing for this position. ScVAF distributions were visually inspected for each variant, and obvious cell doublets (2 mutually exclusive mutations with a scVAF distribution centered on 25%) were manually excluded from the analysis for one patient (PH09). The allelic drop-out (ADO) rate was determined for each sample by Mission Bio pipeline using heterozygous polymorphisms. We then used infSCITE software^2^ (<https://github.com/cbg-ethz/infSCITE>) to infer phylogenetic trees on the resulting mutations matrix for each sample, as already published.^3^ We set false-positive rate argument (-fd) to 1%, false-negative arguments (-ad) to the sample specific ADO rate determined by Mission Bio and the –cc argument to ADO/2*fd. For all samples, infSCITE algorithm retained only one phylogenic tree. The inferred clonal architecture (**Figure S1A**) was used to correct the raw assigned genotype. Cells with insufficient genotype information (sparse genotype) or with a genotype violating the clonal hierarchy owing to cell doublets or sequencing errors (ambiguous genotype) were excluded for downstream analyses (**Figure S1B**). Such phylogeny-driven genotype inference strategy significantly increased the number of cells with retained genotype information for downstream analyses (**Figure S1C**) and was particularly useful when one of the mutations occurred on an amplicon with poor coverage. A homozygous genotype was considered only if the cell fraction involved was > 2 x ADO rate. Protein data was analyzed using Seurat package V4.0.^4^ The single-cell ADT sequencing depth was corrected using a centered log-ratio transformation as recommended for compositional data.^4^ Dimensionality reduction was performed using PCA and UMAP embeddings. Differential ADT expression between clones was performed using ALDEX2 package, which uses a Dirichlet-multinomial model to infer abundance from counts.^5^ Differential abundance testing was done using t tests, p-values were corrected using the Benjamini & Hochberg method and the “Effect” parameter was used to estimate the size of the abundance difference.

**References**

1. Boudry A, Darmon S, Duployez N, Figeac M, Geffroy S, Bucci M*, et al.* Frugal alignment-free identification of FLT3-internal tandem duplications with FiLT3r. *Biorxiv* 2022**:** 2022.2006.2027.496265.

2. Kuipers J, Jahn K, Raphael BJ, Beerenwinkel N. Single-cell sequencing data reveal widespread recurrence and loss of mutational hits in the life histories of tumors. *Genome Res* 2017 Nov; **27**(11)**:** 1885-1894.

3. Morita K, Wang F, Jahn K, Hu T, Tanaka T, Sasaki Y*, et al.* Clonal evolution of acute myeloid leukemia revealed by high-throughput single-cell genomics. *Nat Commun* 2020 Oct 21; **11**(1)**:** 5327.

4. Hao Y, Hao S, Andersen-Nissen E, Mauck WM, 3rd, Zheng S, Butler A*, et al.* Integrated analysis of multimodal single-cell data. *Cell* 2021 Jun 24; **184**(13)**:** 3573-3587 e3529.

5. Fernandes AD, Reid JN, Macklaim JM, McMurrough TA, Edgell DR, Gloor GB. Unifying the analysis of high-throughput sequencing datasets: characterizing RNA-seq, 16S rRNA gene sequencing and selective growth experiments by compositional data analysis. *Microbiome* 2014; **2:** 15.

**Supplementary figure legends.**

**Supplementary Figure S1. A.** Example of a phylogeny tree inferred by infSCITE. **B.** Phylogeny-driven genotype correction strategy. Missing genotypes were inferred using the phylogeny tree. Cells with insufficient genotype information (sparse genotype, orange) or with a genotype violating the clonal hierarchy owing to doublets or PCR errors (ambiguous genotype, red) were excluded from downstream analyses. **C.** Summary of the number of cells detected by the Tapestri pipeline V2 (white), including those with complete raw genotype information (blue), the retained cells with complete genotype after phylogeny-driven correction (green) and those excluded owing to sparse (orange) or ambiguous (red) genotypes.

**Supplementary Figure S2. A.** Correlation between variant allelic frequency (VAF) on bulk HTS and pseudo-bulk VAF calculated on ADT-seq data. Points are colored according to the sample. Only mutations detected by both methods are displayed. Comparison was done using Spearman correlation. The two homozygous mutations are highlighted. **B.** Copy-number of the *FLT3* locus inferred on bulk HTS (Viscap, upper panel) and sc-DNAseq data (lower panel). **C.** Copy-number of the *TET2* locus inferred on bulk HTS (Viscap, upper panel) and sc-DNAseq data (lower panel). **D.** Fishplots showing inferred clonal architecture and clone abundances in the 11 *NPM1*-mutated AML samples.

**Supplementary Figure S3.** Uniform manifold approximation and projection (UMAP) plot of ADT-seq expression of the single-cells from the each of the11 AML samples. Cells are colored according to the retained genotype.

**Supplementary Figure S1.**

**
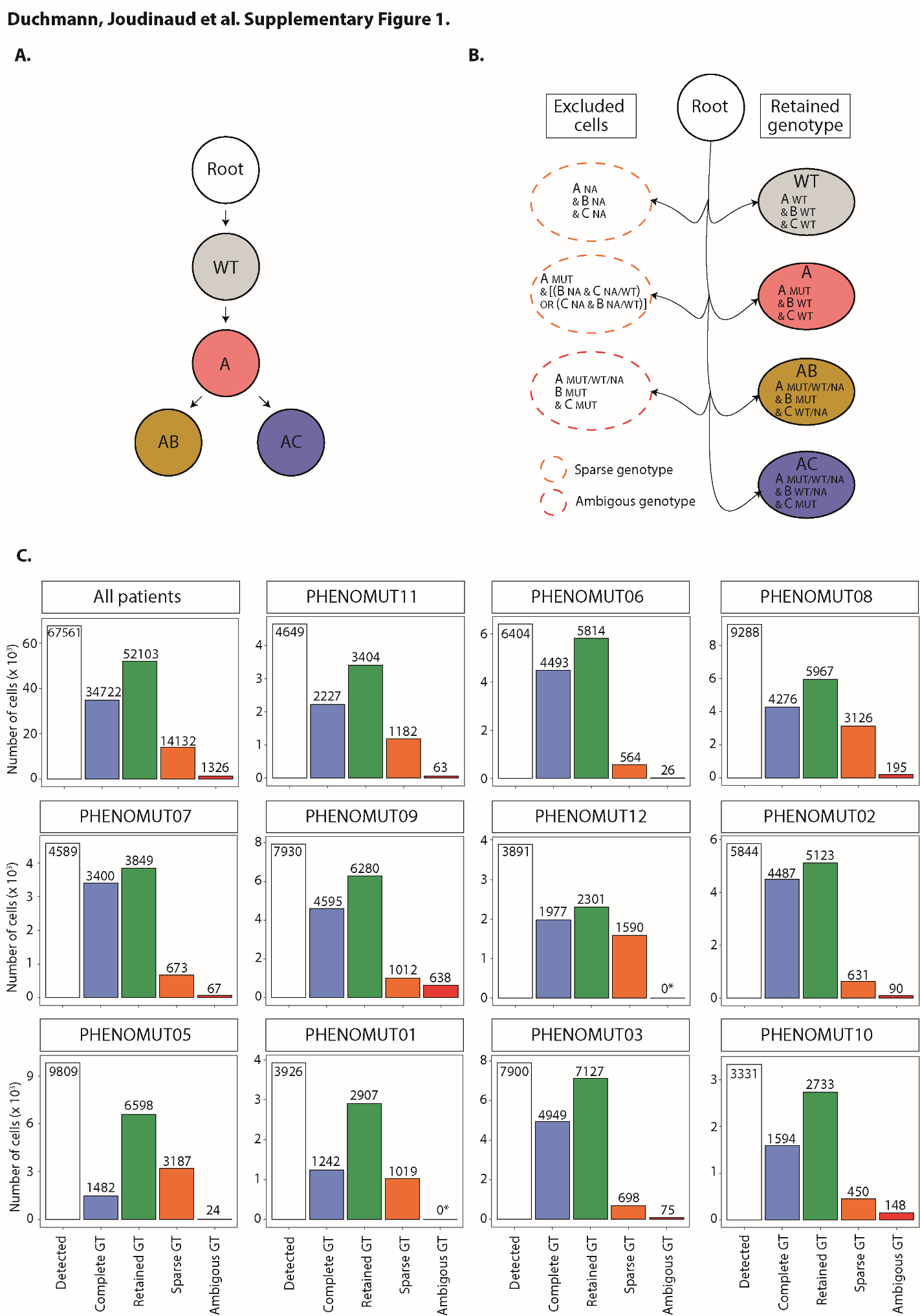
**

**Supplementary Figure S2.**

**
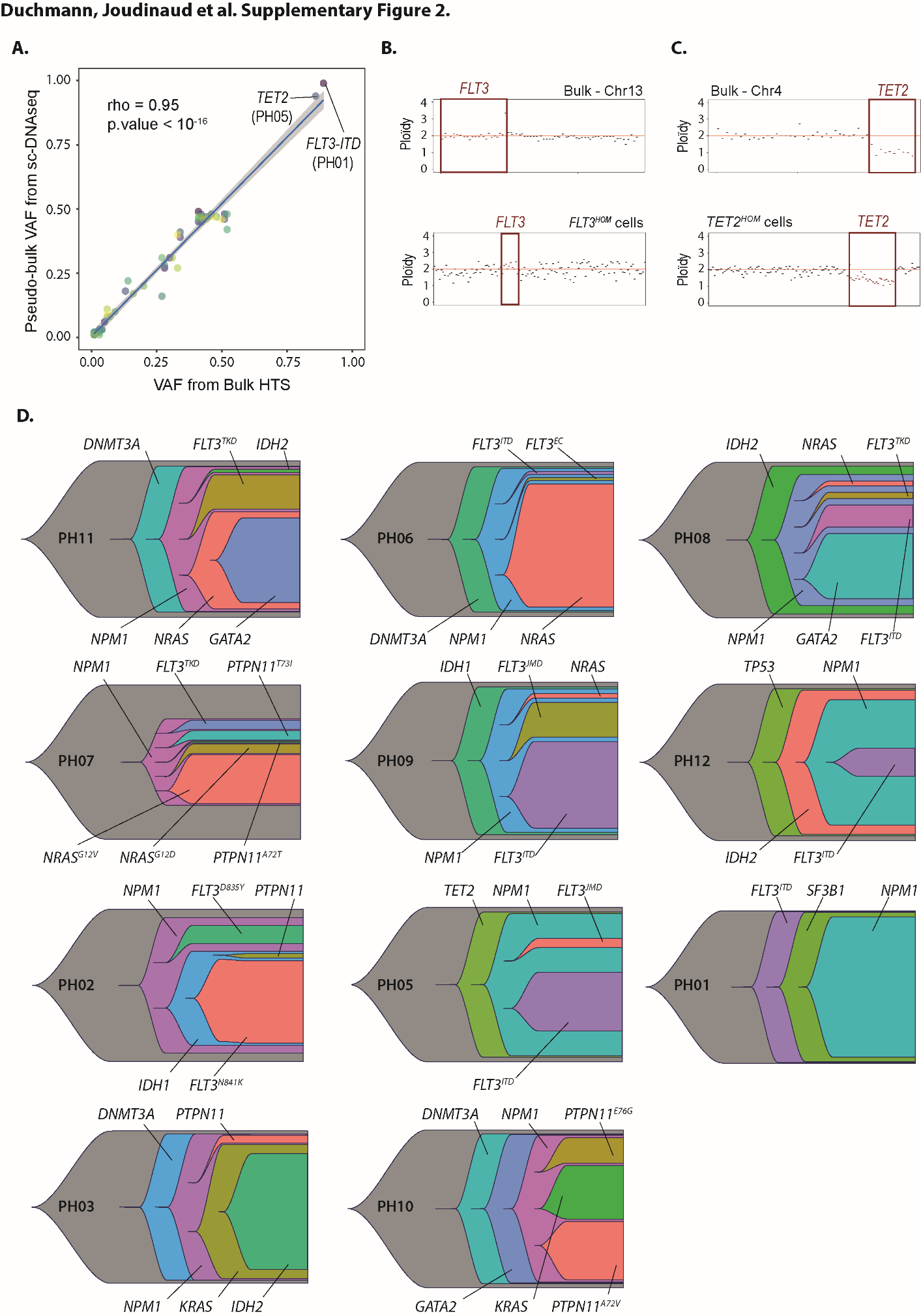
**

**Supplementary Figure S3.**


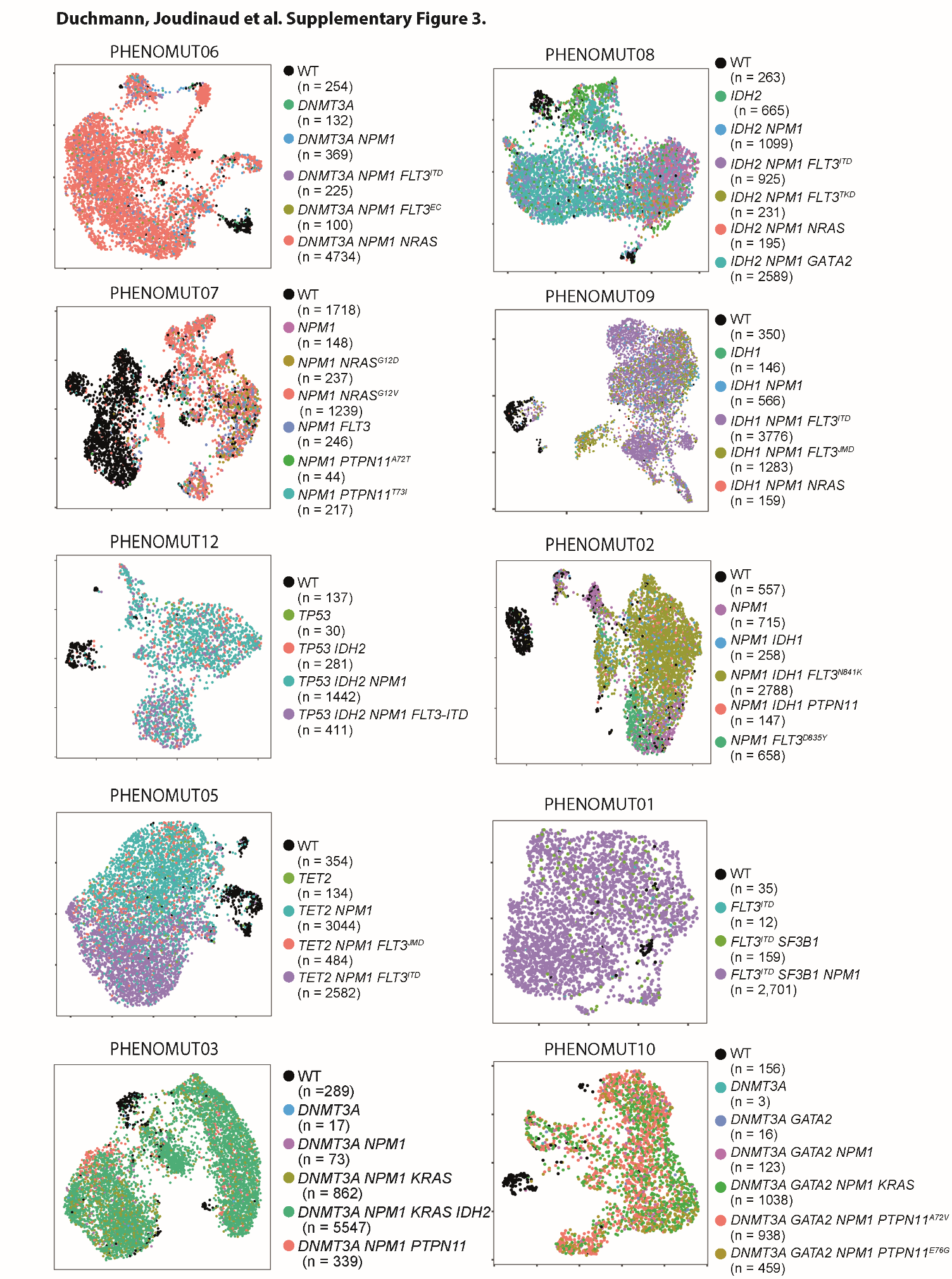


| **Supplementary Table 1. Overlapping genes covered in patients sequenced by Targeted HTS in St Louis hospital** | | |
| --- | --- | --- |
| **Gene** | **Reference Sequence** | **Coverage** |
| *ASXL1* | NM_015338 | all exons |
| *ASXL2* | NM_018263 | all exons |
| *ATRX* | NM_000489 | all exons |
| *BCOR* | NM_017745 | all exons |
| *BCORL1* | NM_021946 | all exons |
| *BRAF* | NM_004333 | all exons |
| *BRCA2* | NM_000059 | all exons |
| *BRCC3* | NM_024332 | all exons |
| *CALR* | NM_004343 | all exons |
| *CBL* | NM_005188 | all exons |
| *CEBPA* | NM_004364 | all exons |
| *CREBBP* | NM_004380 | all exons |
| *CSF3R* | NM_156039 | all exons |
| *CTCF* | NM_006565 | all exons |
| *CUX1* | NM_001913 | all exons |
| *DDX41* | NM_016222 | all exons |
| *DNMT3A* | NM_022552 | all exons |
| *EP300* | NM_001429 | all exons |
| *ERCC6L2* | NM_020207 | all exons |
| *ETNK1* | NM_018638 | all exons |
| *ETV6* | NM_001987 | all exons |
| *EZH2* | NM_004456 | all exons |
| *FLT3* | NM_004119 | all exons |
| *GATA2* | NM_032638 | all exons |
| *IDH1* | NM_005896 | all exons |
| *IDH2* | NM_002168 | all exons |
| *JAK2* | NM_004972 | all exons |
| *KDM6A* | NM_021140 | all exons |
| *KIT* | NM_000222 | all exons |
| *KMT2A* | NM_001197104 | all exons |
| *KMT2D* | NM_003482 | all exons |
| *KRAS* | NM_033360 | all exons |
| *MECOM* | NM_001105078 | all exons |
| *MPL* | NM_005373 | all exons |
| *NF1* | NM_001042492 | all exons |
| *NPM1* | NM_002520 | all exons |
| *NRAS* | NM_002524 | all exons |
| *PHF6* | NM_001015877 | all exons |
| *PRPF8* | NM_006445 | all exons |
| *PTPN11* | NM_002834 | all exons |
| *RAD21* | NM_006265 | all exons |
| *RIT1* | NM_006912 | all exons |
| *RUNX1* | NM_001001890 | all exons |
| *SAMD9* | NM_017654 | all exons |
| *SAMD9L* | NM_152703 | all exons |
| *SETBP1* | NM_015559 | all exons |
| *SF3B1* | NM_012433 | all exons |
| *SMC1A* | NM_006306 | all exons |
| *SMC3* | NM_005445 | all exons |
| *SRP72* | NM_006947 | all exons |
| *SRSF2* | NM_003016 | all exons |
| *STAG2* | NM_001042749 | all exons |
| *TET2* | NM_001127208 | all exons |
| *TP53* | NM_001126112 | all exons |
| *U2AF1* | NM_006758 | all exons |
| *WT1* | NM_024426 | all exons |
| *ZRSR2* | NM_005089 | all exons |

| **Supplementary Table 2. Genes covered in patients sequenced by Targeted HTS in Lille university hospital** | | |
| --- | --- | --- |
| **Gene** | **Reference Sequence** | **Coverage** |
| *ANKRD26* | NM_014915 | 5'UTR |
| *ASXL1* | NM_015338 | Exons 11-12 |
| *ASXL2* | NM_018263 | Exons 11-12 |
| *ATRX* | NM_000489 | all exons |
| *BCOR* | NM_001123385 | all exons |
| *BCORL1* | NM_021946 | all exons |
| *BRAF* | NM_004333 | Exons 11 and 15 |
| *CALR* | NM_004343 | Exon 9 |
| *CBL* | NM_005188 | Exons 8 and 9 |
| *CEBPA* | NM_004364 | Exon 1 |
| *CRLF2* | NM_022148 | Exon 6 |
| *CSF3R* | NM_156039 | Exons 14 and 17 |
| *CUX1* | NM_181552 | all exons |
| *DDX41* | NM_016222 | all exons |
| *DNMT3A* | NM_022552 | all exons |
| *ETNK1* | NM_018638 | all exons |
| *ETV6* | NM_001987 | all exons |
| *EZH2* | NM_004456 | all exons |
| *FLT3* | NM_004119 | all exons |
| *GATA1* | NM_002049 | Exons 2 and 3 |
| *GATA2* | NM_032638 | Exons 2-6 and Intron 4 |
| *GNAS* | NM_000516 | Exons 8 and 9 |
| *GNB1* | NM_002074 | Exons 5 and 6 |
| *HRAS* | NM_005343 | Exon 2-4 |
| *IDH1* | NM_005896 | all exons |
| *IDH2* | NM_002168 | all exons |
| *IKZF1* | NM_006060 | all exons |
| *IL2RG* | NM_000206 | Exon 8 |
| *IL7R* | NM_002185 | Exon 6 |
| *JAK1* | NM_002227 | all exons |
| *JAK2* | NM_004972 | all exons |
| *JAK3* | NM_000215 | all exons |
| *KDM6A* | NM_001291415 | all exons |
| *KIT* | NM_000222 | Exons 8-11 and 17 |
| *KRAS* | NM_033360 | Exons 2-4 |
| *MPL* | NM_005373 | all exons |
| *NF1* | NM_001042492 | all exons |
| *NFE2* | NM_001136023 | all exons |
| *NPM1* | NM_002520 | Exons 10-11 |
| *NRAS* | NM_002524 | Exons 2-4 |
| *PAX5* | NM_016734 | all exons |
| *PHF6* | NM_001015877 | all exons |
| *PPM1D* | NM_003620 | all exons |
| *PTPN11* | NM_002834 | all exons |
| *RAD21* | NM_006265 | all exons |
| *RIT1* | NM_006912 | Exon 5 |
| *RUNX1* | NM_001754 | all exons |
| *SAMD9* | NM_017654 | all exons |
| *SAMD9L* | NM_152703 | all exons |
| *SETBP1* | NM_015559 | all exons |
| *SF3B1* | NM_012433 | Exons 13-16 |
| *SH2B3* | NM_005475 | all exons |
| *SMC1A* | NM_006306 | all exons |
| *SMC3* | NM_005445 | all exons |
| *SRP72* | NM_006947 | all exons |
| *SRSF2* | NM_003016 | Exon 1 |
| *STAG2* | NM_001042749 | all exons |
| *STAT3* | NM_003150 | all exons |
| *STAT5B* | NM_012448 | all exons |
| *TERC* | NR_001566 | all exons |
| *TERT* | NM_198253 | all exons |
| *TET2* | NM_001127208 | all exons |
| *TP53* | NM_001126112 | all exons |
| *U2AF1* | NM_006758 | Exons 2 and 6 |
| *UBA1* | NM_003334 | Exon 3 |
| *WT1* | NM_024426 | all exons |
| *ZRSR2* | NM_005089 | all exons |

| **Supplementary Table 3. List of conjugated antibodies with their respecive DNA Oligos** | | |
| --- | --- | --- |
| **Antibody** | **DNA-tag** | **Titration** |
| CD34 | CAGCCAGAA | 1/2 |
| CD117 | GACTGACAA | 1 |
| CD33 | AACACCGTT | 1/3 |
| CD123 | GCAATGAGT | 1 |
| HLA-DR | GCCTGTTGA | 1/3 |
| CD13 | CCTTAGACC | 1 |
| CD19 | CCTCTATGC | 1 |
| CD38 | CCAACACTA | 1/5 |
| CD11b | CCTGGAATC | 1 |
| CD45 | TCGTGCCAA | 1/2 |
| CD90 | AACGGACGT | 1 |
| CD45RA | CCTTAAGGT | 1 |
| CD56 | AACGCCTCA | 1 |
| CD81 | AACCGAACA | 1/2 |
| CD7 | CAGTAACCT | 1 |

| **Supplementary Table 4. List of genomic DNA amplicons (panel AML v2, Mission Bio°)** | | | | |
| --- | --- | --- | --- | --- |
| hg19 | chr1 | 115256487 | 115256723 | AML_v2_NRAS_115256512 |
| hg19 | chr1 | 115258609 | 115258825 | AML_v2_NRAS_115258635 |
| hg19 | chr2 | 25457143 | 25457372 | AML_v2_DNMT3A_25457166 |
| hg19 | chr2 | 25458518 | 25458763 | AML_v2_DNMT3A_25458540 |
| hg19 | chr2 | 25459793 | 25460046 | AML_v2_DNMT3A_25459813 |
| hg19 | chr2 | 25461880 | 25462137 | AML_v2_DNMT3A_25461902 |
| hg19 | chr2 | 25463106 | 25463346 | AML_v2_DNMT3A_25463127 |
| hg19 | chr2 | 25463493 | 25463717 | AML_v2_DNMT3A_25463515 |
| hg19 | chr2 | 25464421 | 25464618 | AML_v2_DNMT3A_25464443 |
| hg19 | chr2 | 25466621 | 25466871 | AML_v2_DNMT3A_25466642 |
| hg19 | chr2 | 25467012 | 25467220 | AML_v2_DNMT3A_25467033 |
| hg19 | chr2 | 25467371 | 25467631 | AML_v2_DNMT3A_25467391 |
| hg19 | chr2 | 25468110 | 25468332 | AML_v2_DNMT3A_25468130 |
| hg19 | chr2 | 25469006 | 25469231 | AML_v2_DNMT3A_25469026 |
| hg19 | chr2 | 25469407 | 25469606 | AML_v2_DNMT3A_25469428 |
| hg19 | chr2 | 25469925 | 25470185 | AML_v2_DNMT3A_25469945 |
| hg19 | chr2 | 25470403 | 25470663 | AML_v2_DNMT3A_25470423 |
| hg19 | chr2 | 25470930 | 25471190 | AML_v2_DNMT3A_25470951 |
| hg19 | chr2 | 25472515 | 25472728 | AML_v2_DNMT3A_25472538 |
| hg19 | chr2 | 198266103 | 198266322 | AML_v2_SF3B1_198266130 |
| hg19 | chr2 | 198266442 | 198266688 | AML_v2_SF3B1_198266472 |
| hg19 | chr2 | 198266693 | 198266913 | AML_v2_SF3B1_198266718 |
| hg19 | chr2 | 198267319 | 198267569 | AML_v2_SF3B1_198267340 |
| hg19 | chr2 | 209113085 | 209113297 | AML_v2_IDH1_209113110 |
| hg19 | chr3 | 128200077 | 128200327 | AML_v2_GATA2_128200102 |
| hg19 | chr3 | 128200668 | 128200928 | AML_v2_GATA2_128200689 |
| hg19 | chr3 | 128202699 | 128202899 | AML_v2_GATA2_128202719 |
| hg19 | chr4 | 55561579 | 55561792 | AML_v2_KIT_55561605 |
| hg19 | chr4 | 55569875 | 55570095 | AML_v2_KIT_55569899 |
| hg19 | chr4 | 55592077 | 55592282 | AML_v2_KIT_55592099 |
| hg19 | chr4 | 55593509 | 55593744 | AML_v2_KIT_55593532 |
| hg19 | chr4 | 55593963 | 55594183 | AML_v2_KIT_55593987 |
| hg19 | chr4 | 55599270 | 55599486 | AML_v2_KIT_55599293 |
| hg19 | chr4 | 55602651 | 55602862 | AML_v2_KIT_55602675 |
| hg19 | chr4 | 106154923 | 106155158 | AML_v2_TET2_106154951 |
| hg19 | chr4 | 106155158 | 106155416 | AML_v2_TET2_106155178 |
| hg19 | chr4 | 106155469 | 106155729 | AML_v2_TET2_106155489 |
| hg19 | chr4 | 106155913 | 106156173 | AML_v2_TET2_106155932 |
| hg19 | chr4 | 106156237 | 106156489 | AML_v2_TET2_106156259 |
| hg19 | chr4 | 106156503 | 106156762 | AML_v2_TET2_106156523 |
| hg19 | chr4 | 106156793 | 106157046 | AML_v2_TET2_106156812 |
| hg19 | chr4 | 106157077 | 106157332 | AML_v2_TET2_106157098 |
| hg19 | chr4 | 106157426 | 106157679 | AML_v2_TET2_106157447 |
| hg19 | chr4 | 106157756 | 106158015 | AML_v2_TET2_106157777 |
| hg19 | chr4 | 106158029 | 106158288 | AML_v2_TET2_106158049 |
| hg19 | chr4 | 106158293 | 106158544 | AML_v2_TET2_106158314 |
| hg19 | chr4 | 106158545 | 106158805 | AML_v2_TET2_106158576 |
| hg19 | chr4 | 106162388 | 106162618 | AML_v2_TET2_106162415 |
| hg19 | chr4 | 106163952 | 106164192 | AML_v2_TET2_106163979 |
| hg19 | chr4 | 106164705 | 106164952 | AML_v2_TET2_106164725 |
| hg19 | chr4 | 106180718 | 106180956 | AML_v2_TET2_106180745 |
| hg19 | chr4 | 106182829 | 106183072 | AML_v2_TET2_106182858 |
| hg19 | chr4 | 106190733 | 106190955 | AML_v2_TET2_106190758 |
| hg19 | chr4 | 106193540 | 106193777 | AML_v2_TET2_106193572 |
| hg19 | chr4 | 106193777 | 106194032 | AML_v2_TET2_106193797 |
| hg19 | chr4 | 106194035 | 106194295 | AML_v2_TET2_106194057 |
| hg19 | chr4 | 106196179 | 106196429 | AML_v2_TET2_106196202 |
| hg19 | chr4 | 106196437 | 106196692 | AML_v2_TET2_106196456 |
| hg19 | chr4 | 106196771 | 106197024 | AML_v2_TET2_106196791 |
| hg19 | chr4 | 106197028 | 106197278 | AML_v2_TET2_106197048 |
| hg19 | chr4 | 106197335 | 106197593 | AML_v2_TET2_106197355 |
| hg19 | chr4 | 106197598 | 106197858 | AML_v2_TET2_106197618 |
| hg19 | chr5 | 170837384 | 170837659 | AML_v2_NPM1_170837412 |
| hg19 | chr7 | 148504721 | 148504971 | AML_v2_EZH2_148504743 |
| hg19 | chr7 | 148506025 | 148506265 | AML_v2_EZH2_148506050 |
| hg19 | chr7 | 148506371 | 148506589 | AML_v2_EZH2_148506394 |
| hg19 | chr7 | 148507404 | 148507618 | AML_v2_EZH2_148507427 |
| hg19 | chr7 | 148508696 | 148508930 | AML_v2_EZH2_148508719 |
| hg19 | chr7 | 148511032 | 148511276 | AML_v2_EZH2_148511054 |
| hg19 | chr7 | 148511990 | 148512230 | AML_v2_EZH2_148512017 |
| hg19 | chr7 | 148514917 | 148515124 | AML_v2_EZH2_148514941 |
| hg19 | chr7 | 148523626 | 148523866 | AML_v2_EZH2_148523652 |
| hg19 | chr7 | 148525653 | 148525888 | AML_v2_EZH2_148525675 |
| hg19 | chr7 | 148526737 | 148526948 | AML_v2_EZH2_148526763 |
| hg19 | chr7 | 148529630 | 148529890 | AML_v2_EZH2_148529658 |
| hg19 | chr7 | 148543467 | 148543693 | AML_v2_EZH2_148543492 |
| hg19 | chr7 | 148544266 | 148544493 | AML_v2_EZH2_148544293 |
| hg19 | chr9 | 5073698 | 5073902 | AML_v2_JAK2_5073725 |
| hg19 | chr11 | 32413427 | 32413633 | AML_v2_WT1_32413452 |
| hg19 | chr11 | 32414185 | 32414405 | AML_v2_WT1_32414209 |
| hg19 | chr11 | 32417758 | 32417989 | AML_v2_WT1_32417780 |
| hg19 | chr11 | 32421511 | 32421750 | AML_v2_WT1_32421532 |
| hg19 | chr11 | 32439082 | 32439321 | AML_v2_WT1_32439105 |
| hg19 | chr12 | 25378534 | 25378794 | AML_v2_KRAS_25378559 |
| hg19 | chr12 | 25380238 | 25380478 | AML_v2_KRAS_25380260 |
| hg19 | chr12 | 25398206 | 25398433 | AML_v2_KRAS_25398232 |
| hg19 | chr12 | 112888115 | 112888350 | AML_v2_PTPN11_112888140 |
| hg19 | chr12 | 112890994 | 112891234 | AML_v2_PTPN11_112891019 |
| hg19 | chr12 | 112910667 | 112910907 | AML_v2_PTPN11_112910689 |
| hg19 | chr12 | 112915377 | 112915582 | AML_v2_PTPN11_112915401 |
| hg19 | chr12 | 112924188 | 112924405 | AML_v2_PTPN11_112924215 |
| hg19 | chr12 | 112926203 | 112926424 | AML_v2_PTPN11_112926226 |
| hg19 | chr12 | 112926824 | 112927050 | AML_v2_PTPN11_112926847 |
| hg19 | chr13 | 28589756 | 28589977 | AML_v2_FLT3_28589783 |
| hg19 | chr13 | 28592473 | 28592726 | AML_v2_FLT3_28592494 |
| hg19 | chr13 | 28597497 | 28597727 | AML_v2_FLT3_28597520 |
| hg19 | chr13 | 28601130 | 28601358 | AML_v2_FLT3_28601153 |
| hg19 | chr13 | 28602302 | 28602559 | AML_v2_FLT3_28602324 |
| hg19 | chr13 | 28608188 | 28608395 | AML_v2_FLT3_28608210 |
| hg19 | chr13 | 28608473 | 28608696 | AML_v2_FLT3_28608497 |
| hg19 | chr13 | 28609571 | 28609790 | AML_v2_FLT3_28609594 |
| hg19 | chr13 | 28610014 | 28610260 | AML_v2_FLT3_28610043 |
| hg19 | chr15 | 90631740 | 90631990 | AML_v2_IDH2_90631760 |
| hg19 | chr17 | 7572906 | 7573129 | AML_v2_TP53_7572930 |
| hg19 | chr17 | 7573973 | 7574178 | AML_v2_TP53_7573996 |
| hg19 | chr17 | 7576759 | 7576976 | AML_v2_TP53_7576782 |
| hg19 | chr17 | 7577014 | 7577264 | AML_v2_TP53_7577035 |
| hg19 | chr17 | 7577397 | 7577636 | AML_v2_TP53_7577424 |
| hg19 | chr17 | 7578075 | 7578315 | AML_v2_TP53_7578098 |
| hg19 | chr17 | 7578362 | 7578618 | AML_v2_TP53_7578383 |
| hg19 | chr17 | 7579858 | 7580118 | AML_v2_TP53_7579878 |
| hg19 | chr17 | 74732191 | 74732450 | AML_v2_SRSF2_74732219 |
| hg19 | chr20 | 30956749 | 30956969 | AML_v2_ASXL1_30956774 |
| hg19 | chr20 | 31015813 | 31016051 | AML_v2_ASXL1_31015840 |
| hg19 | chr20 | 31021137 | 31021366 | AML_v2_ASXL1_31021160 |
| hg19 | chr20 | 31021438 | 31021659 | AML_v2_ASXL1_31021460 |
| hg19 | chr20 | 31022168 | 31022417 | AML_v2_ASXL1_31022192 |
| hg19 | chr20 | 31022719 | 31022978 | AML_v2_ASXL1_31022741 |
| hg19 | chr20 | 31023008 | 31023248 | AML_v2_ASXL1_31023032 |
| hg19 | chr20 | 31023261 | 31023492 | AML_v2_ASXL1_31023285 |
| hg19 | chr20 | 31023556 | 31023761 | AML_v2_ASXL1_31023578 |
| hg19 | chr21 | 36171567 | 36171811 | AML_v2_RUNX1_36171592 |
| hg19 | chr21 | 36206683 | 36206913 | AML_v2_RUNX1_36206703 |
| hg19 | chr21 | 36231692 | 36231937 | AML_v2_RUNX1_36231714 |
| hg19 | chr21 | 36252819 | 36253046 | AML_v2_RUNX1_36252844 |
| hg19 | chr21 | 44514549 | 44514808 | AML_v2_U2AF1_44514570 |
| hg19 | chr21 | 44524416 | 44524634 | AML_v2_U2AF1_44524438 |

| **Supplementary Table 5. Patient characteristics.** | | | | | | | | | | | | | | |
| --- | --- | --- | --- | --- | --- | --- | --- | --- | --- | --- | --- | --- | --- | --- |
| **Patient** | **Center** | **Age** | **Sex** | **WBC**  **(x10^9^/L)** | **PB**  **Blasts** | **BM**  **Blasts** | **FAB** | **Karyotype** | **Mutations**  **covered by MB** | **Not**  **Covered** | **Sample**  **site** | **Treatement** | **Response** | **Relapse** |
| PHENOMUT01 | St Louis | 60 y.o | F | 39 | 47% | 93% | LAM5 | Normal | *SF3B1, NPM1, FLT3-ITD* | None | BM | 3+7 | CR | No |
| PHENOMUT02 | Lille | 47 y.o | F | 13 | 62% | 75% | LAM2 | Normal | *NPM1, IDH1, FLT3-TKD x2, PTPN11* | None | BM | 3+7+Midostaurine | CR | No |
| PHENOMUT03 | St Louis | 44 y.o | F | 88 | 73% | 87% | LAM4 | Normal | *DNMT3A, NPM1, KRAS, IDH2, PTPN11* | *RAD21* | BM | 3+7 | CR | No |
| PHENOMUT05 | St Louis | 88 y.o | F | 90 | 90% | 96% | LAM1 | Normal | *TET2, NPM1, FLT3-ITD, FLT3-JMD* | *SRSF2* | BM | Sorafenib | PD | NA |
| PHENOMUT06 | St Louis | 43 y.o | M | 59 | 8% | 60% | LAM4 | Normal | *DNMT3A, NPM1, NRAS, FLT3-ITD, FLT3-EC* | None | BM | 3+7 | CR | No |
| PHENOMUT07 | Lille | 24 y.o | M | 32 | 10% | 23% | LAM5 | Normal | *NPM1, NRAS x 2, FLT3-TKD, PTPN11 x 2* | None | BM | 3+7+Midostaurine | CR | Yes |
| PHENOMUT08 | St Louis | 79 y.o | M | 75 | 14% | 75% | LAM1 | Normal | *GATA2, FLT3-ITD, NRAS, FLT3-TKD* | *SRSF2* | BM | AZA | PD | NA |
| PHENOMUT09 | St Louis | 35 y.o | F | 81 | 94% | 92% | LAM1 | Normal | *IDH1, NPM1, FLT3-ITD, FLT3-JMD, NRAS* | None | BM | 3+7+Midostaurine | CR | No |
| PHENOMUT10 | St Louis | 50 y.o | M | 61 | 42% | 84% | LAM5 | Normal | *DNMT3A, NPM1, GATA2, KRAS, PTPN11 x 2* | None | BM | 3+7 | CR | No |
| PHENOMUT11 | St Louis | 68 y.o | M | 32 | 6% | 26% | LAM5 | Normal | *DNMT3A, NPM1, GATA2, NRAS, FLT3-TKD, IDH2, NFE2* | *SMC3, NFE2* | BM | 3+7 | CR | No |
| PHENOMUT12 | St Louis | 62 y.o | M | 123 | 86% | 96% | LAM1 | Normal | *IDH2, TP53, NPM1, FLT3-ITD* | *FLT3-ITD* | PB | 3+7+Midostaurine | PD | NA |

| **Supplementary Table 6. Variants detected by bulk HTS** | | | |  |  |
| --- | --- | --- | --- | --- | --- |
| **Patient** | **Gene** | **Variant** | **VAF** | **Covered** | **Detected** |
| PHENOMUT01 | *FLT3* | FLT3_ITD21 | 0.89 | Yes | Yes |
| PHENOMUT01 | *NPM1* | NPM1_p.L287fs | 0.43 | Yes | Yes |
| PHENOMUT01 | *SF3B1* | SF3B1_p.K666N | 0.41 | Yes | Yes |
| PHENOMUT02 | *NPM1* | NPM1_p.W288fs | 0.42 | Yes | Yes |
| PHENOMUT02 | *FLT3* | FLT3_p.N841K | 0.28 | Yes | Yes |
| PHENOMUT02 | *FLT3* | FLT3_p.D835Y | 0.05 | Yes | Yes |
| PHENOMUT02 | *PTPN11* | PTPN11_p.D61G | 0.01 | Yes | Yes |
| PHENOMUT02 | *IDH1* | IDH1_p.R132H | 0.3 | Yes | Yes |
| PHENOMUT03 | *IDH2* | IDH2_p.R140Q | 0.34 | Yes | Yes |
| PHENOMUT03 | *KRAS* | KRAS_p.G12C | 0.41 | Yes | Yes |
| PHENOMUT03 | *DNMT3A* | DNMT3A_p.567del | 0.42 | Yes | Yes |
| PHENOMUT03 | *NPM1* | NPM1_p.L287fs | 0.51 | Yes | Yes |
| PHENOMUT03 | *PTPN11* | PTPN11_p.A72T | 0.02 | Yes | Yes |
| PHENOMUT03 | *RAD21* | RAD21_p.R338X | 0.43 | **No** | **No** |
| PHENOMUT05 | *TET2* | TET2_p.M695fs | 0.86 | Yes | Yes |
| PHENOMUT05 | *NPM1* | NPM1_p.L287fs | 0.52 | Yes | Yes |
| PHENOMUT05 | *SRSF2* | SRSF2_p.P95H | 0.4 | **No** | **No** |
| PHENOMUT05 | *FLT3* | FLT3_ITD21 | 0.18 | Yes | Yes |
| PHENOMUT05 | *FLT3* | FLT3_p.F594C | 0.03 | Yes | Yes |
| PHENOMUT06 | *DNMT3A* | DNMT3A_p.R882H | 0.46 | Yes | Yes |
| PHENOMUT06 | *NPM1* | NPM1_p.L287fs | 0.44 | Yes | Yes |
| PHENOMUT06 | *NRAS* | NRAS_p.G12V | 0.33 | Yes | Yes |
| PHENOMUT06 | *FLT3* | FLT3_ITD27 | 0.01 | Yes | Yes |
| PHENOMUT06 | *FLT3* | FLT3_p.V491L | 0.01 | Yes | Yes |
| PHENOMUT07 | *NPM1* | NPM1_p.L287fs | 0.28 | Yes | Yes |
| PHENOMUT07 | *FLT3* | FLT3_p.D835Y | 0.04 | Yes | Yes |
| PHENOMUT07 | *NRAS* | NRAS_p.G12V | 0.27 | Yes | Yes |
| PHENOMUT07 | *NRAS* | NRAS_p.G12D | 0.04 | Yes | Yes |
| PHENOMUT07 | *PTPN11* | PTPN11_p.T73I | 0.03 | Yes | Yes |
| PHENOMUT07 | *PTPN11* | PTPN11_p.A72T | 0.01 | Yes | Yes |
| PHENOMUT08 | *NPM1* | NPM1_p.L287fs | 0.52 | Yes | Yes |
| PHENOMUT08 | *IDH2* | IDH2_p.R140Q | 0.52 | Yes | Yes |
| PHENOMUT08 | *SRSF2* | SRSF2_p.P95H | 0.49 | **No** | **No** |
| PHENOMUT08 | *GATA2* | GATA2_p.A372T | 0.14 | Yes | Yes |
| PHENOMUT08 | *NRAS* | NRAS_p.G12D | 0.03 | Yes | Yes |
| PHENOMUT08 | *FLT3* | FLT3_ITD54 | 0.06 | Yes | Yes |
| PHENOMUT08 | *FLT3* | FLT3_D835Y | 0.01 | Yes | Yes |
| PHENOMUT09 | *IDH1* | IDH1_p.R132G | 0.48 | Yes | Yes |
| PHENOMUT09 | *FLT3* | FLT3_ITD39 | 0.27 | Yes | Yes |
| PHENOMUT09 | *FLT3* | FLT3_p.V592A | 0.09 | Yes | Yes |
| PHENOMUT09 | *NPM1* | NPM1_p.W288fs | 0.45 | Yes | Yes |
| PHENOMUT09 | *NRAS* | NRAS_p.G13V | 0.03 | Yes | Yes |
| PHENOMUT10 | *PTPN11* | PTPN11_p.E76G | 0.07 | Yes | Yes |
| PHENOMUT10 | *PTPN11* | PTPN11_p.A72V | 0.16 | Yes | Yes |
| PHENOMUT10 | *KRAS* | KRAS_p.G13D | 0.2 | Yes | Yes |
| PHENOMUT10 | *DNMT3A* | DNMT3A_p.R882H | 0.45 | Yes | Yes |
| PHENOMUT10 | *NPM1* | NPM1_p.L287fs | 0.42 | Yes | Yes |
| PHENOMUT10 | *GATA2* | GATA2_p.R307L | 0.41 | Yes | Yes |
| PHENOMUT11 | *SMC3* | SMC3_p.382_387del | 0.08 | **No** | **No** |
| PHENOMUT11 | *GATA2* | GATA2_p.R362Q | 0.33 | Yes | Yes |
| PHENOMUT11 | *NRAS* | NRAS_p.G12D | 0.31 | Yes | Yes |
| PHENOMUT11 | *NPM1* | NPM1_p.L287fs | 0.43 | Yes | Yes |
| PHENOMUT11 | *FLT3* | FLT3_p.A680V | 0.06 | Yes | Yes |
| PHENOMUT11 | *DNMT3A* | DNMT3A_p.N879D | 0.44 | Yes | Yes |
| PHENOMUT11 | *IDH2* | IDH2_p.R140Q | 0.01 | Yes | Yes |
| PHENOMUT11 | *NFE2* | NFE2_p.R312fs | 0.09 | **No** | **No** |
| PHENOMUT12 | *IDH2* | IDH2_p.R140Q | 0.5 | Yes | Yes |
| PHENOMUT12 | *FLT3* | FLT3_ITD102 | 0.11 | Yes | **No** |
| PHENOMUT12 | *TP53* | TP53_p.R267L | 0.47 | Yes | Yes |
| PHENOMUT12 | *NPM1* | NPM1_p.L287fs | 0.33 | Yes | Yes |
| PHENOMUT12 | *FLT3* | FLT3_ITD73 | 0.06 | Yes | Yes |

| **Supplementary Table 7. Variants detected by bulk HTS on bulk and subfractions of PHENOMUT11.** | | | | |
| --- | --- | --- | --- | --- |
| **Gene** | **Variant** | **VAF in Bulk** | **VAF in Mono-like** | **log2(FC)** |
| *DNMT3A* | DNMT3A_p.N879D | 0.44 | 0.43 | -0.03 |
| *FLT3* | FLT3_p.A680V | **0.06** | **0.40** | **2.74** |
| *GATA2* | GATA2_p.R362Q | **0.33** | **0.01** | **-5.04** |
| *IDH2* | IDH2_p.R140Q | 0.01 | 0.03 | 1.58 |
| *NFE2* | NFE2_p.R312fs | **0.09** | **0.47** | **2.38** |
| *NPM1* | NPM1_p.L287fs | 0.43 | 0.47 | 0.13 |
| *NRAS* | NRAS_p.G12D | **0.31** | **0.01** | **-4.95** |
| *SMC3* | SMC3_p.382_387del | **0.08** | **0.44** | **2.46** |
